# Who complies with COVID-19 transmission mitigation behavioral guidelines?

**DOI:** 10.1371/journal.pone.0240396

**Published:** 2020-10-08

**Authors:** Ahmed Maged Nofal, Gabriella Cacciotti, Nick Lee

**Affiliations:** 1 Emlyon Business School, Ecully, France; 2 Hankamer School of Business, Waco, Texas, United States of America; 3 Warwick Business School, University of Warwick, Coventry, United Kingdom; Middlesex University, UNITED KINGDOM

## Abstract

During the past 6 months, the world has lost almost 950,000 lives because of the outbreak of COVID-19, with more than 31 million individuals diagnosed with COVID-19 worldwide. In response, lockdowns, and various other policies have been implemented. Unfortunately, many individuals are violating those policies and governments have been urging people to comply with the behavioral guidelines. In this paper, we argue that personality traits need to be considered to understand and encourage more effective public compliance with COVID 19 transmission mitigation behavioral guidelines. Using a sample of 8,548 individuals from Japan, we show that certain personality traits are related to the tendency to comply with COVID-19 transmission mitigation behavioral guidelines. We emphasize the importance of understanding why people respond differently to the same authority’s messages and provide actionable insights for government policy makers and those who implement policies.

## Introduction

Since the outbreak of COVID-19, a number of initiatives have been put in place with the objective of mitigating transmission of the virus, such as imposing lockdowns, closing schools, and encouraging social distancing. Medical scientists are racing to find a vaccine. Geographers and software developers are developing applications to track people’s movements in an attempt to identify and contain the virus in specific regions. Health and Media officials are promoting social distancing and persuading the population to follow health behavioral guidelines, often involving significant behavioral changes. Despite these initiatives, as of mid-2020 the world is still losing thousands of lives every day. In a recent statement, Tedros Adhanom Ghebreyesus, director-general of the World Health Organization, stated that “although many countries have made some progress, globally, the pandemic is actually speeding up” [1]. Therefore, there is a clear need to explore further ways to help mitigate the spread of COVID-19. In this regard, “social and behavioral science can provide valuable insights for managing the pandemic and its impact” [2, p. 1], by enhancing our understanding of the role of human behavior in the spread of the virus, and thus contribute to the public good.

While the COVID-19 pandemic has resulted in the development and implementation of numerous behavioral guidelines intended to mitigate transmission, evidence suggests that many of these guidelines are not being followed by enough people to make them optimally effective [3]. Governments and health authorities are still pleading for individuals to behave responsibly, by complying with these imposed behavioral guidelines and rules [4, 5]. However, laboratory studies show that “persuasive appeals are more effective in influencing behavior when they are tailored to individuals’ unique psychological characteristics” [6, p. 12714]. In simple terms, what convinces one person to behave in a particular way, might not do so for another. Moreover, some people are by nature more likely than others to be rule-breakers [7]. For instance, evidence shows that emotionally unstable [8], and disagreeable people [9, 10] are more likely to break rules in general. Accordingly, while it is important to understand which behaviors influence the speed of spread of COVID-19 [11], it is also important to understand the specific personality characteristics that could plausibly be related to the propensity of the population to comply with COVID-19 transmission mitigation behavioral guidelines.

To examine this, we investigate whether certain personality traits are related to compliance with transmission mitigation behavioral guidelines. Particularly, we examine if major personality traits (i.e. conscientiousness, agreeableness, openness to experience, extraversion, and emotional stability) [12], yield differences in the tendency of people to comply with 21 implemented COVID-19 transmission mitigation behavioral guidelines (see S1 Appendix).

This paper makes a number of contributions. First, it draws attention to the importance of individuals’ personalities when assessing the likelihood that an outbreak of the virus will occur among some people but not others. In this regard, we respond to research questions raised by Davidai, Day [13] regarding who complies with COVID-19 transmission mititgation behavioral guidelines. Second, this is not the first crisis the world has faced and will, unfortunately, not be the last. Thus, our work provides evidence of how different personality traits can help people face the outbreak of crises such as global pandemics, and emphasizes the importance of implementation measures which take account of individuals’ personality traits. In fact, this should help to ensure that we learn important lessons from the crisis, so that the responses of governments and organizations will be more effective when future crises occur [11].

Third and most importantly, to our knowledge, the paper provides the first empirical evidence using an exogenous shock (i.e. the COVID-19 outbreak) of the importance of personality traits and motivational propensities when monitoring people’s tendency to comply with COVID-19 transmission mitigation behavioral guidelines. In fact, there has been work exploring the effect of COVID-19-related messages on people’s actual compliance with COVID-19 transmission mitigation behavioral guidelines [14–16], research showing gender differences with regards to the compliance of transmission mitigation behavioral guidelines [17], and studies examining the effect of emotional responses to pro-social messages on the tendency of people to comply with self-isolation restrictions [18]. However, there has been no work examining the influence of personality traits, such as the big five (i.e. conscientiousness, agreeableness, openness to experience, extraversion, and emotional stability) on the tendency of people to comply with COVID-19 transmission mitigation behavioral guidelines. Connecting literatures of emotional responses to COVID-19 messages, and personalized/tailored communication, we believe that this research not only supports prior work in highlighting the importance of pro-social messages, but also the importance of knowing the specific personality traits that may make individuals less likely to comply with COVID-19 transmission mitigation behavioral guidelines.

Hence, the aim of this paper is threefold. First, drawing from research on persuasive mass communication [e.g., 19], we aim to demonstrate if personality traits, specifically, conscientiousness, agreeableness, openness to experience, extraversion, and emotional stability, relate to the tendency of people to comply with COVID-19 transmission mitigation behavioral guidelines. Second, we highlight the importance of incorporating behavioral science into governments, and organizations’ responses to crises, such as COVID-19. Third and most importantly, we aim to provide some practical insights, such as occupational targeting and the use of social network platforms, such as Facebook, Twitter, and Linkedin, to minimize the risk of not complying with COVID-19 transmission mititgation behavioral guidelines.

## Persuasive mass communication, personality traits, and compliance with COVID-19 behavioral guidelines

In psychology, persuasive mass communication refers to the process by which large groups of individuals are encouraged to adopt beliefs, attitudes and behaviors that are consistent with the communicator’s viewpoint [6]. Persuasive mass communication theories aim, thus, to identify strategies to improve the effectiveness of persuasive mass communication messages [e.g. 20, 21]. One such theory, psychological persuasion, assumes that persuasive communication is most effective “when tailored to people’s unique psychological characteristics and motivations” [6, p. 12714]. This means that messages that are more congruent with an individual’s motivational tendencies are processed more smoothly and assessed more positively than incongruent messages [22–24]. Unfortunately, in some situations where mass communication messages are needed, such as COVID-19 transmission mitigation behavioral guidelines, it is often impractical to make the messages either personalized or individually-targeted. In these situations, it becomes even more important to understand how individual differences in motivational tendencies result in differences in the attitudes and behaviors that individuals may adopt in response to the same message [25, 26]. Because motivational tendencies are strongly influenced by personality traits [27], the latter can yield differences in beliefs, attitudes and/or behaviors even when individuals are exposed to the same mass communication message. In this respect, the behavior or the attitude towards the same authority’s messages (as in the case of COVID-19) can be more/less pronounced among people who possess certain personality traits.

Therefore, building on this literature, we consider how different personalities respond differently to the same message from authorities, and hence comply with behavioral guidelines contained in those messages. To do so, we use the Big Five model of personality which relies on the assumption that individual differences are best captured by five global traits [12, 28]: extraversion, emotional stability, openness to experience, agreeableness, and conscientiousness. Each personality trait reflects a different motivational system that influences how individuals perceive the context around them, process authority’s messages, and in turn decide to adopt (or not) compliance behavior. We explore these differences and build our hypotheses next.

### Extraversion

Extraversion refers to the extent to which individuals are assertive, dominant, energetic, active, talkative, impulsive, and enthusiastic [12]. Individuals who score high on extraversion tend to be cheerful, are positively disposed towards other people and large groups, and seek excitement and external stimulation. Meanwhile, individuals who score low on extraversion prefer to spend more time alone and can be described as reserved, quiet, and independent [29]. In this sense, messages containing requests to respect self-isolation and social distancing are incongruent with a motivational system that gains rewards and satisfaction from active engagement with the world, social interaction and social recognition [30]. Accordingly, such messages are not processed smoothly and effectively which results in a tendency to violate such policy measures.

*Hypothesis 1*: There is a negative association between extraversion and the tendency of people to comply with COVID-19 transmission mitigation behavioral guidelines.

### Emotional stability

Emotionally unstable individuals are especially sensitive to threats and uncertainty [31, 32]. Individuals who score low on emotional stability tend to experience a number of negative emotions such as anxiety, depression, hostility, self-consciousness, and vulnerability [12]. Individuals who score high on emotional stability can be described as self-confident, calm, and relaxed [29]. These psychological characteristics suggest that messages containing rules and policies aiming at providing structures and formalized procedures are positively accepted by a motivational system that is normally inhibited by uncertainty and unpredictability [31, 33]. However, enacting self-isolation and keeping social distancing for long periods of time can result in increased stress and negative emotions for emotionally unstable individuals, than for more emotionally stable individuals. This could possibly motivate the former to break rules as an attempt to protect their psychological wellbeing [8]. As such, while individuals who score low on emotional stability can respond to authorities’ messages in different ways, we assume that the opportunity of feeling secure by complying with rules and policies will not be perceived as such if it results in increased risk of anxiety and depression. Accordingly, such messages are not processed fluently and effectively which leads to a tendency for those lower in emotional stability to violate authorities’ rules and behavioral guidelines.

*Hypothesis 2*: There is a positive association between emotional stability and the tendency of people to comply with COVID-19 transmission mitigation behavioral guidelines.

### Openness to experience

Openness to experience is a personality trait that characterizes someone who values creativity, innovation, and intellectual stimulation [34]. Individuals who score high on openness to experience can show active imagination, creativity, preference for variety, and intellectual curiosity. Individuals who score low on openness to experience can be described as conventional, narrow in interests, and unanalytical. These psychological characteristics suggest that open individuals can perceive the self-isolation and social distancing rules as an opportunity to cultivate their curiosity and imagination. The lockdown can create the conditions (e.g., by increasing time for themselves) to engage in activities that promote absorption, self-examination, and creative solutions [12]. Accordingly, they have little interest in escaping a situation that allows to satisfy their needs for intellectual stimulation and cognition [35].

*Hypothesis 3*: There is a positive association between openness to experience and the tendency of people to comply with COVID-19 transmission mitigation behavioral guidelines.

### Agreeableness

Agreeable individuals value communal goals and interpersonal harmony [36]. People who score high on agreeableness have strong cooperative values and a preference for positive interpersonal relationships [9, 10]. People who score low on agreeableness can be described as manipulative, self-centered, suspicious, and aggressive [12, 28, 29]. Because agreeableness may lead one to be particularly empathetic, individuals at the high end of this personality construct tend to show great concern for the welfare of others [36]. Accordingly, they are likely to be very motivated to comply with rules and behavioral guidelines that present self-isolation and social distancing as a way to protect themselves and those around them.

*Hypothesis 4*: There is a positive association between agreeableness and the tendency of people to comply with COVID-19 transmission mitigation behavioral guidelines.

### Conscientiousness

Conscientious individuals value achievement, order, hard work, and efficiency [37, 38]. Individuals high on conscientiousness are described as careful and diligent as opposed to easy going and disorderly [39]. The psychological characteristics of conscientious individuals suggest that violation of rules and behavioral guidelines aiming at controlling social mobility to limit the spread of the virus is not consistent with their tendency and motivation to show self-discipline, act dutifully, and achieve efficiency [12, 40].

*Hypothesis 5*: There is a positive association between conscientiousness and the tendency of people to comply with COVID-19 transmission mitigation behavioral guidelines.

## Material and methods

### Participants

Our sample is drawn from Japanese citizens aged 20 to 64 years old. The data was collected between the 26^th^ and 28^th^ of March 2020. A sample of 11,342 individuals responded to the survey. A quota sampling approach was adopted to match the sample distribution to the Japanese population in terms of gender, age, and employment status. After excluding individuals who did not respond to any of the items measuring our variables of interests, missing values, and individuals who responded with “don’t know” to any of our variables of interest, we were left with a sample of 8,548 individuals. Further information on the study is available at https://www.openicpsr.org/openicpsr/project/118584/version/V1/view. It is also worth-noting that this research does not require an Ethics Committee approval because it is a secondary analysis of anonymous data.

### Measures

#### Dependent variable

Adoption of Transmission Mitigation behavioral guidelines. The measure of our outcome variable is measured using policy statements that assess the extent to which individuals adopt the transmission mitigation behavioral guidelines. The data includes 21 behavioral guidelines developed to prevent the spread of the virus, such as respondents’ tendency to “always wear a surgical-style mask when going out”, “stockpile surgical-style masks”, “avoid large gatherings”, and “participate in virtual events using online tools” (see S1 Appendix for the full scale items). For each policy, respondents selected the extent to which they adopted the policy on a 5-point scale; 1- Very true, 2- True, 3- Neither, 4- Not true, and 5- Not at all. We reversed the scale so that a higher score corresponds to a more likelihood of compliance with the transmission mitigation behavioral guidelines. Internal consistency analysis of the measure returned a Cronbach’s alpha value of .89.

#### Independent variables

*Personality traits*. We use a short scale, very well-established in personality research, to assess individuals’ psychological traits, specifically the Ten Item Personality Inventory (TIPI) [41, p. 289, 42, 43]. The TIPI is comprised of 10 items, each consisting of a pair of traits (see S1 Appendix). For consistency of interpretation, we reverse some responses, such that the higher the value, the higher the level of extraversion, openness to experience, agreeableness, conscientiousness, and emotional stability. The Cronbach’s alpha value of each of the big five ranged from 0.3 to 0.6, which is a limitation that we consider below.

#### Control variables

We control for the effects of a number of covariates. Specifically, We control for the influences of gender (1- male, 2- female) because research shows that gender is related to various psychological traits [44]. Further, as socio-economic conditions could have an effect on both personality traits, and individual behaviors [45], we control for the dummies of individuals’ annual household income, with the first dummy corresponding to having “less than 2,000 K Japanese Yen”, and the highest dummy corresponding to “more than 20,000K JPY” (see S1 Appendix for further details). Following prior research on the relationship between the big five and education [46], we also account for the influence of education by controlling for whether respondents are university or college graduates. Furthermore, as age captures various factors that could influence individuals’ personality traits [47, 48], we control for age categories as dummies. We also control for marital status (1- married, 2- unmarried) because marital status might confound the effects of the big five on different behaviors [48, 49]. In addition, having a partner might impact certain behavioral guidelines, such as gathering with friends. Furthermore, because the sources of information used by individuals may affect the tendency of people to comply with the COVID-19 transmission mitigation behavioral guidelines, we control for the sources of information, such as “TV news programs”, “Information sent by the Ministry of Health, Labor and Welfare”, and “Information sent by local (prefecture) government” (see S1 Appendix for further details). All our sample is drawn from Japan, and therefore location is automatically controlled for.

## Statistical analyses and results

We use Stata MP version 16 for the analyses. Table 1 presents the correlations between the variables (see S1 Table in S1 Appendix for further details). Our sample consists entirely of Japanese citizens. Approximately 54% of the sample are males and 46% are females. In terms of marital status, approximately 38% of the sample are married and 62% are not married. The sample includes various age groups ranging from the age of 20 to the age of 64 years old.

**Table 1:** Correlations.

	1	2	3	4	5	6	7	8	9	10	11
1. Adoption of Transmission Mitigation behavioral guidelines	1.00										
2. Extraversion	0.08*	1.00									
3. Agreeableness	0.11*	-0.00	1.00								
4. Conscientiousness	0.14*	0.22*	0.23*	1.00							
5. Openness to Experience	0.10*	0.37*	0.06*	0.25*	1.00						
6. Emotional Stability	0.04*	0.26*	0.27*	0.35*	0.27*	1.00					
7. Sex	0.13*	0.10*	0.06*	-0.01	-0.12*	-0.12*	1.00				
8. Age	0.04*	0.02*	0.12*	0.15*	-0.01	0.15*	0.00	1.00			
9. Marital Status	0.11*	0.12*	0.09*	0.09*	0.00	0.08*	0.03*	0.27*	1.00		
10. Education	-0.04*	-0.02	-0.02	-0.09*	-0.06*	-0.08*	0.10*	0.02	-0.03*	1.00	
11. Household Income	0.08*	0.15*	0.07*	0.15*	0.08*	0.18*	-0.10*	0.11*	0.31*	-0.23*	1.00

Note: Sample Size = 8,548,

* p<0.05.

Table 2 presents the results of a number of OLS models examining the influence of personality traits on the compliance with COVID-19 transmission mitigation behavioral guidelines. Specifically, model 1 examines the effect of personality traits on the compliance with COVID-19 transmission mitigation behavioral guidelines without any controls. We find support for all our hypotheses apart from extraversion which supports a significant positive effect on compliance with COVID-19 transmission mitigation behavioral guidelines. Model 2 adds our control variables, and again we find support for our hypotheses. Importantly, when adding controls in Model 2, we now find that extraversion negatively influences the tendency of people to comply with COVID-19 transmission mitigation behavioral guidelines, meanwhile agreeableness, conscientiousness, and openness to experience positively influence the tendency of people to comply with COVID-19 transmission mitigation behavioral guidelines. However, our results show that emotional stability does not affect compliance with COVID-19 transmission mitigation behavioral guidelines.

**Table 2:** The effect of personality traits on the compliance with COVID-19 behavioral guidelines [55].

Outcome: Compliance with behavioral guidelines	Model 1	Model 2
Extraversion	0.04***	-0.02**
	(0.01)	(0.01)
Agreeableness	0.09***	0.04***
	(0.01)	(0.01)
Conscientiousness	0.11***	0.09***
	(0.01)	(0.01)
Openness to Experience	0.05***	0.04***
	(0.01)	(0.01)
Emotional Stability	-0.05***	-0.01
	(0.01)	(0.01)
Sex		0.15***
		(0.01)
Marital Status		0.07***
		(0.01)
Education		-0.02
		(0.01)
Information: TV News		-0.02***
		(0.01)
Information: TV Shows		-0.02***
		(0.01)
Information: Newspapers		-0.01***
		(0.00)
Information: Tabloid Paper		-0.05***
		(0.01)
Information: Internet		-0.05***
		(0.01)
Information: SNS App News		-0.03***
		(0.00)
Information: Prime Minister		-0.00
		(0.01)
Information: Ministry of Health		-0.04***
		(0.01)
Information: Government Meetings		-0.04***
		(0.01)
Information: Local Government		-0.06***
		(0.01)
Age Dummies		Included
Household Income Dummies		Included
Multilevel Estimator	Not Applied	Not Applied
Constant	2.97***	3.81***
	(0.04)	(0.07)
Sample Size	10,682	8,548
R-squared	0.04	0.19

Robust standard errors in parentheses.

*** p<0.01,

** p<0.05,

* p<0.1.

While we are cautious about causality issues given the cross-sectional nature of our data, we emphasize that COVID-19 transmission mitigation behavioral guidelines are a result of an exogenous shock (i.e. the sudden outbreak of COVID-19). As Dunning [50] explains, exogenous shocks constitute natural experiments in which subjects are-as-if-randomly assigned to different levels of a treatment and therefore may allow researchers to uncover causal relationships. Therefore, causality issues are reduced. However, despite our inclusion of a number of key controls, omitted variables might still bias the estimates of our model parameters. One way to deal with these potential biases is the use of multilevel modeling [51, 52]. As such, we perform an additional multi-level analysis to overcome the aforementioned issues of omitted variable bias. The rationale for applying a multilevel estimator is the fact that for a global phenomenon as the COVID-19 pandemic, government communications have often been aimed at certain specific societal groups, including different age groups [53], and household characteristics [54]. For instance, older people are asked to be more cautious compared to younger ones [53]. Therefore, multilevel models can be used to capture variations at both levels; individuals at the first level, as well as age and household annual income groups at the second level. Hence, for robustness reasons, we re-estimate our models using the multilevel estimator we find similar results to the OLS analysis; with and without the control variables (see S1 Appendix).

**Fig 1:**
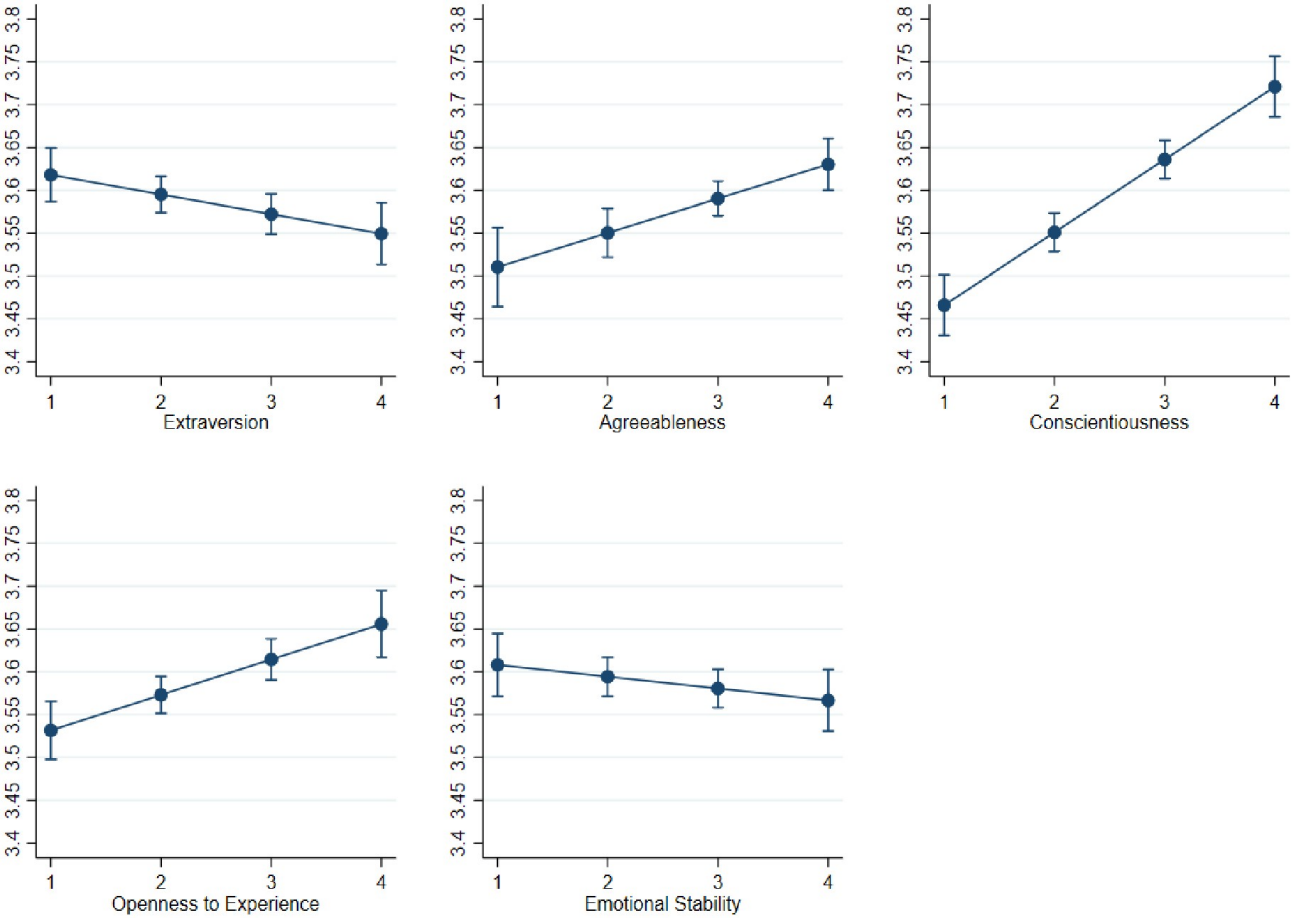
The effect size of personality traits on individuals’ compliance with COVID-19 behavioral guidelines.

Because our large sample size increases our power to detect statistical significance of even small effects, we also consider the substantive importance of our results in terms of effect size, using the odds ratio. Specifically, our findings indicate that the highest odds ratio is for conscientiousness, with people high in conscientiousness 31% more likely to adopt COVID-19 transmission mitigation behavioral guidelines compared to people low in conscientiousness, followed by 19% for openness to experience, and 17% for agreeableness (i.e. these percentages are calculated based on the odds ratios computed based on a 1 unit increase/decrease on the scale items used). We also found that people high in extraversion are 7% less likely to adopt COVID-19 transmission mitigation behavioral guidelines compared to introverts (see Fig 1). It is worth mentioning that our outcome is assessed using scale items and therefore the linear coefficients estimated might overestimate/underestimate the effect size of our independent variables [56, 57]. Therefore, we have calculated the effect sizes based on odds ratios estimated through ordinal logit models (see also Fig 1 for linear effect sizes). In this respect, we emphasize that in the case of an ordinal outcome such as the scale item being used, the interpretation is not read as a contrast between extremes as in the binary outcome case, but as a contrast for each unit change. In other words, on a scale demarcated as 1, 2, 3, 4, 5 as in the case of our compliance measure, an OR of 1.1, for instance, entails a 10% increase in the odds of the dependent variable for each unit change in the independent variable (e.g. moving from 1 to 2, or 2 to 3, or 3 to 4, or 4 to 5 on the scale demarcated). For robustness, we re-test our hypotheses after splitting our sample into males and females. We find consistent results across both samples for agreeableness, conscientiousness, and openness to experience. However, extraversion shows a negative influence on the adoption of COVID-19 transmission mitigation behavioral guidelines only for the male sample, but not for the female sample.

## Discussion

The COVID-19 pandemic has resulted in the introduction of an unprecedented number of governmental policy measures intended to contain the health crisis and mitigate its effects on the economy [58]. However, as of mid-2020, most governments are still struggling to contain the outbreak of COVID-19. In this paper, we argue that the effectiveness of governmental messaging, in terms of its influence on behavioral change of individual citizens, is dependent on individual differences in personality traits. There are a number of key implications of our findings, which translate into actionable insights for government policy makers and those who implement policies. A first key implication is that governments need to tailor messaging towards different personality traits. At the top of the list in this regard is conscientiousness. In essence, using a sample of 8,549 individuals from Japan, we find that conscientiousness increases the likelihood of adopting COVID-19 transmission mitigation behavioral guidelines by 31%. In fact, identifying individuals low in conscientiousness can be helpful in knowing the communities where transmission mitigation behavioral guidelines are least likely to be adopted. Though we do not find consistent patterns for other personality traits, we find tentative evidence of the role of extraversion, agreeableness, and openness to experience in the adoption COVID-19 transmission mitigation behavioral guidelines, with the highest effect size detected for openness to experience, followed by agreeableness, and then extraversion.

We emphasize that these effect sizes are not deterministic. Personality influences imply probabilistic propensities rather than hard-wired patterns of COVID-19 guidelines compliance behavior. Thus, our suggestion that the influence of our independent variables on the tendency of people to comply with COVID-19 transmission mitigation behavioral guidelines should not be confounded with determinism. That said, when dealing with a societal-level communication task such as that required for pandemic-mitigation guidelines, understanding these probabilistic propensities is highly valuable, even if they are not deterministic *per se*.

Our study provides several contributions. To begin with, the value of our findings can be seen in the example of efforts to increase the number of people who comply with COVID-19 transmission mitigation behavioral guidelines. This can be accomplished by implementing initiatives that can help trigger some personality traits, including relatively static ones [59, 60]. For instance, since our findings show that conscientiousness considerably increases the likelihood that people adopt COVID-19 transmission mitigation behavioral guidelines, governments are encouraged to boost citizens’ sense of belonging and obligation to their communities–which has been suggested to develop conscientiousness [61, 62]. Moreover, because our findings show that openness to experience, especially for males, positively influences the tendency of people to adopt transmission mitigation behavioral guidelines, promoting online inductive reasoning training can be beneficial for developing openness to experience [63] and therefore the adoption of transmission mitigation behavioral guidelines. In addition, as our results indicate that agreeableness positively influences the adoption of transmission mitigation behavioral guidelines, especially for females, governments are urged to promote training on balancing criticism and empathy to trigger higher levels of agreeableness [64] and hence higher adoption of behavioral guidelines.

Further, researchers argue that contact tracing is of significant importance to contain the spread of the virus [65]. Yet, many countries are still struggling to develop effective ways of tracing. Given our findings, we have three suggestions. First, countries can trace groups that are less likely to have developed high levels of conscientiousness, openness to experience, and agreeableness. One quite feasible way of doing this is through sending out short surveys on personality traits using social network platforms, such as Facebook, Twitter, and Linkedin. Based on those surveys, governments and organizations can provide different COVID-19 messages. Another way is through occupational categorizing, for instance, entrepreneurs are known for their high levels of conscientiousness, and openness to experience, unlike full-time employees [27, 49]. Hence, tracing certain occupational groups (i.e. entrepreneurs and full-time employees) might be informative in this respect (indeed, we empirically examined this suggestion and found that fulltime employees are less likely to comply with COVID-19 transmission mitigation behavioral guidelines compared to entrepreneurs, part-time employees, and unemployed individuals). As a result, governments can send different messages to different occupational groups and therefore increase the likelihood that those occupational groups comply with the announced COVID-19 transmission mitigation behavioral guidelines.

Second, assessing individuals’ personalities could be beneficial to identify people who tend to violate the transmission mitigation behavioral guidelines. For instance, using the TIPI which is an “ultra-short scale” [41, p. 289], countries can collect information about the people who tend not to adopt the COVID-19 transmission mitigation behavioral guidelines. Further, recent research shows that digital footprints, such as their Facebook Likes or Tweets, can predict people’s personality traits [6]. Thus, observing individuals’ digital footprints could inform about their personalities, and therefore help us identify the people who are least likely to adopt the transmission mitigation behavioral guidelines. That said, we recognise that the privacy implications of such initiatives would also have to be balanced with their potential effectiveness.

In sum, with our study, we respond to the question of Davidai, Day [13, p. 3] on who complies with COVID-19 transmission mitigation behavioral guidelines. Based on our findings, we provide one plausible answer, that is: those who are high in conscientiousness, introverts, and agreeable people as well as individuals who are high in openness to experience. We also provide insights for COVID-19 media campaigns. We demonstrate the potential importance of tailoring campaigns to people’s psychological characteristics–which may help provide an explanation for why the significant resources that have been expended on campaigns to encourage transmission mitigation behaviors have yielded sometimes little effect.

This research has some limitations. First, the TIPI that is used to measure the Big Five might be inferior to other scales, given its internal-consistency issues [27]. However, as Park, Wiernik [66, p. 1] explain, different big-five factor models are recommended based on “specific studies, research questions, and contexts”. As the TIPI is characterized by having a high level of content validity and is highly urged in situations “when brevity is a priority” [41, p. 289], such as the outbreak of the deadly pandemic, we see its relevance to our study. Meanwhile, future research is urged to retest our hypotheses using other scales.

Second, although our findings can be insightful to many countries, our study applies to Japan. Countries have implemented to a great extent the same COVID-19 transmission mitigation behavioral guidelines, yet there have been some differences. Even proximate countries showed different reactions to COVID-19. For example, Sweden has implemented very few behavioral guidelines to face the pandemic unlike its neighboring Finland which has implemented stricter quarantine conditions. Therefore, further research focusing on other countries is urged.

Third, the small amount of variance explained by the big five (4%) suggests that the big five personality traits alone do not completely predict compliance with COVID-19 transmission mitigation behavioral guidelines. Rather, they may play a role in one of the many mechanisms explaining compliance with COVID-19 transmission mitigation behavioral guidelines. Therefore, it is important for future research to examine different interactive effects of the big five with other factors on compliance with COVID-19 transmission mitigation behavioral guidelines [67].

## Conclusion

Over 100 years ago, Science magazine published an article that put down some lessons from the Spanish Flu pandemic [68]. As Bavel, Baicker [2] explain, the paper demonstrates that three main factors contribute to the outspread of a pandemic: (1) individuals do not appreciate the risks they undertake, (2) people are resistant to shutting themselves up in strict isolation as a means to contain the outbreak of a pandemic, and (3) people in many instances act as a continuing danger to others and themselves unconsciously. Therefore, “how individuals respond to advice on how best to prevent transmission will be as important as government actions, if not more important” [65, p. 934]. A century later, in the midst of another pandemic, we hope the present paper helps provide some assistance in this regard.

## Supporting information

S1 Appendix (DOCX)
