# Supplementary material for: Who complies with COVID-19 transmission mitigation behavioral guidelines?
Source: PLoS One. 2020 Oct 8;15(10):e0240396. doi: 10.1371/journal.pone.0240396 (PMC7544078; doi:10.1371/journal.pone.0240396)
Supplement: S1 Appendix — (DOCX) [file pone.0240396.s001.docx]

**Online Appendix**

**COVID-19 transmission mitigation policies:** The 21 policies are: 1- avoid a poorly-ventilated closed space, 2- avoid large gatherings, 3- avoid conversations or shouting in close proximity, 4- avoid places where items 1, 2, and 3 overlap, 5- do not go to dinner with friends, 6- do not go to mass gatherings, 7- participate in virtual events using online tools, 8- undertake frequent hand washing, 9- undertake cough etiquette, 10- disinfect things around, 11- avoid going out when you have a cold, 12- avoid going to clinic even when having a cold symptom, 13- prepare consultation and transportation methods for when you feel ill, 14- always wear a surgical-style mask when going out, 15- stockpile surgical-style mask, 16- stockpile food, toilet paper, tissue paper, etc., 17- avoid contact with younger people, 18- avoid contact with older people, 19- get sufficient rest and sleep, 20- eat a nutritious diet, and 21- do exercise such as jogging or exercise using DVD.

**TIPI measure of Personality traits:** 1- Extraverted, enthusiastic, 2- Critical, quarrelsome, 3- Dependable, self-disciplined, 4- Anxious, easily upset, 5- Open to new experiences, complex, 6- Reserved, quiet, 7- Sympathetic, warm, 8- Disorganized, careless, 9- Calm, emotionally stable, and 10- Conventional, uncreative [47]. Extraversion is assessed using items 1 and 6, agreeableness is assessed using items 2 and 7, conscientiousness is assessed using items 3 and 8, openness to experience is assessed using item 5 and 10, and emotional stability is assessed using items 4 and 9 on a four point scale (i.e. 1- agree strongly, 2- agree, 3- disagree, and 4- disagree strongly).

**Measures of Controls:**

**Household income:** 1- less than 2,000K Japanese Yen (JPY), 2- 2,000-3,999K JPY, 3- 4,000-5,999K JPY, 4- 6,000-6,999K JPY, 5- 8,000-8,999K JPY, 6- 10,000-11,999K JPY, 7- 12,000-14,999K JPY, 8- 15,000-19,999K JPY, 9- More than 20,000K JPY, and 10- Do not know.

We dropped individuals who responded with “do not know” and included the dummies of all other categories

**Sources of Informations:** 1- TV news programs, 2- TV talk and variety shows, 3- Newspapers (national and local newspapers), 4- Tabloid paper, 5- Internet news sites, 6- SNS app news, 7- Information sent by the Prime Minister, 8- Information sent by the Ministry of Health, Labor and Welfare, 9- Information provided by government Expert Meetings, and 10- Information sent by local (prefecture) government. We control for the dummies of all these categories.

Table S1

*Detailed Correlations and Descriptive Data*

|  | 1 | 2 | 3 | 4 | 5 | 6 | 8 | 9 | 10 | 11 | 12 | 13 | 14 | 15 | 16 | 17 | 18 | 19 | 20 | 21 | 22 |
| --- | --- | --- | --- | --- | --- | --- | --- | --- | --- | --- | --- | --- | --- | --- | --- | --- | --- | --- | --- | --- | --- |
| 1. Number of Policies Adopted | 1.00 |  |  |  |  |  |  |  |  |  |  |  |  |  |  |  |  |  |  |  |  |
| 1. Extraversion | 0.08* | 1.00 |  |  |  |  |  |  |  |  |  |  |  |  |  |  |  |  |  |  |  |
| 1. Agreeableness | 0.11* | -0.00 | 1.00 |  |  |  |  |  |  |  |  |  |  |  |  |  |  |  |  |  |  |
| 1. Conscientiousness | 0.14* | 0.22* | 0.23* | 1.00 |  |  |  |  |  |  |  |  |  |  |  |  |  |  |  |  |  |
| 1. Openness to Experience | 0.10* | 0.37* | 0.06* | 0.25* | 1.00 |  |  |  |  |  |  |  |  |  |  |  |  |  |  |  |  |
| 1. Emotional Stability | 0.04* | 0.26* | 0.27* | 0.35* | 0.27* | 1.00 |  |  |  |  |  |  |  |  |  |  |  |  |  |  |  |
| 1. Sex | 0.13* | 0.10* | 0.06* | -0.01 | -0.12* | -0.12* | 1.00 |  |  |  |  |  |  |  |  |  |  |  |  |  |  |
| 1. Age | 0.04* | 0.02* | 0.12* | 0.15* | -0.01 | 0.15* | 0.00 | 1.00 |  |  |  |  |  |  |  |  |  |  |  |  |  |
| 1. Marital Status | 0.11* | 0.12* | 0.09* | 0.09* | 0.00 | 0.08* | 0.03* | 0.27* | 1.00 |  |  |  |  |  |  |  |  |  |  |  |  |
| 1. Education | -0.04* | -0.02 | -0.02 | -0.09* | -0.06* | -0.08* | 0.10* | 0.02 | -0.03* | 1.00 |  |  |  |  |  |  |  |  |  |  |  |
| 1. Household Income | 0.08* | 0.15* | 0.07* | 0.15* | 0.08* | 0.18* | -0.10* | 0.11* | 0.31* | -0.23* | 1.00 |  |  |  |  |  |  |  |  |  |  |
| 1. Information: TV News | -0.19* | -0.08* | -0.13* | -0.03* | 0.01 | -0.01 | -0.11* | -0.14* | -0.15* | -0.01 | -0.08* | 1.00 |  |  |  |  |  |  |  |  |  |
| 1. Information: TV Shows | -0.18* | -0.09* | -0.07* | 0.00 | 0.00 | 0.03* | -0.15* | -0.04* | -0.11* | -0.08* | -0.00 | 0.58* | 1.00 |  |  |  |  |  |  |  |  |
| 1. Information: Newspapers | -0.16* | -0.07* | -0.04* | -0.11* | -0.07* | -0.09* | 0.11* | -0.24* | -0.11* | 0.14* | -0.21* | 0.18* | 0.11* | 1.00 |  |  |  |  |  |  |  |
| 1. Information: Tabloid Paper | -0.15* | -0.08* | 0.09* | -0.05* | -0.11* | -0.04* | 0.22* | 0.05* | -0.00 | 0.04* | -0.07* | -0.03* | 0.11* | 0.37* | 1.00 |  |  |  |  |  |  |
| 1. Information: Internet | -0.20* | -0.02 | -0.12* | -0.01 | -0.03* | 0.00 | -0.03* | -0.04* | -0.04* | 0.06* | -0.08* | 0.25* | 0.14* | 0.09* | -0.00 | 1.00 |  |  |  |  |  |
| 1. Information: SNS App News | -0.19* | -0.09* | -0.00 | 0.05* | -0.10* | 0.05* | -0.02 | 0.24* | 0.03* | 0.02 | -0.01 | 0.06* | 0.15* | 0.06* | 0.25* | 0.27* | 1.00 |  |  |  |  |
| 1. Information: Prime Minister | -0.29* | -0.09* | -0.12* | -0.08* | -0.10* | -0.05* | -0.04* | -0.12* | -0.12* | 0.05* | -0.13* | 0.32* | 0.27* | 0.21* | 0.13* | 0.29* | 0.25* | 1.00 |  |  |  |
| 1. Information: Ministry of Health | -0.31* | -0.10* | -0.12* | -0.08* | -0.11* | -0.06* | -0.04* | -0.12* | -0.10* | 0.05* | -0.12* | 0.30* | 0.26* | 0.22* | 0.14* | 0.28* | 0.24* | 0.85* | 1.00 |  |  |
| 1. Information: Government Meetings | -0.30* | -0.11* | -0.09* | -0.09* | -0.10* | -0.07* | -0.02 | -0.15* | -0.11* | 0.07* | -0.14* | 0.29* | 0.27* | 0.26* | 0.19* | 0.25* | 0.23* | 0.72* | 0.78* | 1.00 |  |
| 1. Information: Local Government | -0.31* | -0.08* | -0.08* | -0.07* | -0.09* | -0.03* | -0.06* | -0.08* | -0.12* | 0.05* | -0.09* | 0.23* | 0.20* | 0.27* | 0.18* | 0.24* | 0.23* | 0.52* | 0.54* | 0.55* | 1.00 |

Note: Sample Size = 8,548, * p<0.05

Table S2

The Effect of Personality Traits on the Compliance with COVID-19 behavioral guidelines using Multilevel Modeling based on Age and Household Income Groups

| Outcome: Compliance with behavioral guidelines | Model 1 | Model 2 | Model 3 | Model 4 |
| --- | --- | --- | --- | --- |
| Extraversion | 0.03*** | -0.02*** | 0.04*** | -0.02** |
|  | (0.01) | (0.01) | (0.01) | (0.01) |
| Agreeableness | 0.09*** | 0.04*** | 0.09*** | 0.04*** |
|  | (0.01) | (0.01) | (0.01) | (0.01) |
| Conscientiousness | 0.10*** | 0.09*** | 0.10*** | 0.08*** |
|  | (0.01) | (0.01) | (0.01) | (0.01) |
| Openness to Experience | 0.06*** | 0.04*** | 0.05*** | 0.04*** |
|  | (0.01) | (0.01) | (0.01) | (0.01) |
| Emotional Stability | -0.05*** | -0.01 | -0.05*** | -0.01 |
|  | (0.01) | (0.01) | (0.01) | (0.01) |
| Sex |  | 0.15*** |  | 0.15*** |
|  |  | (0.01) |  | (0.01) |
| Marital Status |  | 0.07*** |  | 0.07*** |
|  |  | (0.01) |  | (0.01) |
| Education |  | -0.02* |  | -0.02 |
|  |  | (0.01) |  | (0.01) |
| Information: TV News |  | -0.02*** |  | -0.02*** |
|  |  | (0.01) |  | (0.01) |
| Information: TV Shows |  | -0.02*** |  | -0.02*** |
|  |  | (0.01) |  | (0.01) |
| Information: Newspapers |  | -0.01*** |  | -0.01*** |
|  |  | (0.00) |  | (0.00) |
| Information: Tabloid Paper |  | -0.05*** |  | -0.05*** |
|  |  | (0.01) |  | (0.01) |
| Information: Internet |  | -0.05*** |  | -0.05*** |
|  |  | (0.01) |  | (0.01) |
| Information: SNS App News |  | -0.03*** |  | -0.03*** |
|  |  | (0.00) |  | (0.00) |
| Information: Prime Minister |  | -0.00 |  | -0.00 |
|  |  | (0.01) |  | (0.01) |
| Information: Ministry of Health |  | -0.04*** |  | -0.04*** |
|  |  | (0.01) |  | (0.01) |
| Information: Government Meetings |  | -0.03*** |  | -0.03*** |
|  |  | (0.01) |  | (0.01) |
| Information: Local Government |  | -0.06*** |  | -0.06*** |
|  |  | (0.01) |  | (0.01) |
| Age Dummies |  | Included | 2^nd^ Level | 2^nd^ Level |
| Household Income Dummies | 2^nd^ Level | 2^nd^ Level |  | Included |
| Multilevel Estimator | Applied | Applied | Applied | Applied |
| Constant | 2.99*** | 3.77*** | 2.99*** | 3.82*** |
|  | (0.04) | (0.06) | (0.04) | (0.06) |
| Sample Size | 8,063 | 8,548 | 10,682 | 8,548 |
| R-squared | 0.04 | 0.19 | 0.04 | 0.19 |

Table S3

*The Effect of the Big Five Personality Traits on the Adoption of COVID-19 Transmission Mitigation Behavioral Guidelines (Males Only)*

| Outcome: Policies Adopted | Model 1 | Model 2 | Model 3 |
| --- | --- | --- | --- |
| Extraversion | -0.03* | -0.03** | -0.02** |
|  | (0.01) | (0.01) | (0.01) |
| Agreeableness | 0.03** | 0.03** | 0.03** |
|  | (0.02) | (0.01) | (0.01) |
| Conscientiousness | 0.09*** | 0.09*** | 0.09*** |
|  | (0.01) | (0.01) | (0.01) |
| Openness to Experience | 0.05*** | 0.05*** | 0.05*** |
|  | (0.01) | (0.01) | (0.01) |
| Emotional Stability | -0.03* | -0.03* | -0.03* |
|  | (0.02) | (0.01) | (0.01) |
| Marital Status | 0.06*** | 0.05*** | 0.05*** |
|  | (0.02) | (0.02) | (0.02) |
| Education | -0.01 | -0.01 | -0.01 |
|  | (0.02) | (0.02) | (0.02) |
| Information: TV News | -0.03*** | -0.03*** | -0.03*** |
|  | (0.01) | (0.01) | (0.01) |
| Information: TV Shows | -0.02** | -0.02** | -0.02** |
|  | (0.01) | (0.01) | (0.01) |
| Information: Newspapers | -0.01** | -0.01** | -0.01** |
|  | (0.01) | (0.01) | (0.01) |
| Information: Tabloid Paper | -0.05*** | -0.05*** | -0.05*** |
|  | (0.01) | (0.01) | (0.01) |
| Information: Internet | -0.06*** | -0.06*** | -0.06*** |
|  | (0.01) | (0.01) | (0.01) |
| Information: SNS App News | -0.03*** | -0.03*** | -0.03*** |
|  | (0.01) | (0.01) | (0.01) |
| Information: Prime Minister | -0.00 | -0.00 | -0.00 |
|  | (0.01) | (0.01) | (0.01) |
| Information: Ministry of Health | -0.02 | -0.02 | -0.02 |
|  | (0.02) | (0.02) | (0.02) |
| Information: Government Meetings | -0.06*** | -0.06*** | -0.06*** |
|  | (0.01) | (0.01) | (0.01) |
| Information: Local Government | -0.06*** | -0.06*** | -0.06*** |
|  | (0.01) | (0.01) | (0.01) |
| Age Dummies | Included | Included | 2^nd^ Level |
| Household Income Dummies | Included | 2^nd^ Level | Included |
| Multilevel Estimator | Not Applied | Applied | Applied |
| Constant | 4.13*** | 4.07*** | 4.10*** |
|  | (0.09) | (0.08) | (0.08) |
| Sample Size | 4,679 | 4,679 | 4,679 |
| R-squared | 0.20 | 0.20 | 0.20 |

Robust standard errors in parentheses

*** p<0.01, ** p<0.05, * p<0.1

Table S4

*The Effect of the Big Five Personality Traits on the Adoption of COVID-19 Transmission Mitigation Behavioral Guidelines (Females Only)*

| Outcome: Policies Adopted | Model 1 | Model 2 | Model 3 |
| --- | --- | --- | --- |
| Extraversion | -0.02 | -0.02 | -0.02 |
|  | (0.01) | (0.01) | (0.01) |
| Agreeableness | 0.05*** | 0.05*** | 0.05*** |
|  | (0.02) | (0.01) | (0.01) |
| Conscientiousness | 0.08*** | 0.08*** | 0.08*** |
|  | (0.01) | (0.01) | (0.01) |
| Openness to Experience | 0.04*** | 0.04*** | 0.04*** |
|  | (0.01) | (0.01) | (0.01) |
| Emotional Stability | -0.01 | -0.01 | -0.00 |
|  | (0.01) | (0.01) | (0.01) |
| Marital Status | 0.09*** | 0.10*** | 0.10*** |
|  | (0.02) | (0.02) | (0.02) |
| Education | -0.03* | -0.03* | -0.03 |
|  | (0.02) | (0.02) | (0.02) |
| Information: TV News | -0.01 | -0.01 | -0.01 |
|  | (0.01) | (0.01) | (0.01) |
| Information: TV Shows | -0.02* | -0.02* | -0.01* |
|  | (0.01) | (0.01) | (0.01) |
| Information: Newspapers | -0.01 | -0.01 | -0.01* |
|  | (0.01) | (0.01) | (0.01) |
| Information: Tabloid Paper | -0.06*** | -0.06*** | -0.06*** |
|  | (0.01) | (0.01) | (0.01) |
| Information: Internet | -0.03*** | -0.03*** | -0.03*** |
|  | (0.01) | (0.01) | (0.01) |
| Information: SNS App News | -0.02*** | -0.02*** | -0.02*** |
|  | (0.01) | (0.01) | (0.01) |
| Information: Prime Minister | -0.00 | -0.00 | -0.00 |
|  | (0.01) | (0.01) | (0.01) |
| Information: Ministry of Health | -0.06*** | -0.06*** | -0.06*** |
|  | (0.02) | (0.02) | (0.02) |
| Information: Government Meetings | -0.01 | -0.01 | -0.01 |
|  | (0.01) | (0.01) | (0.01) |
| Information: Local Government | -0.06*** | -0.06*** | -0.06*** |
|  | (0.01) | (0.01) | (0.01) |
| Age Dummies | Included | Included | 2^nd^ Level |
| Household Income Dummies | Included | 2^nd^ Level | Included |
| Multilevel Estimator | Not Applied | Applied | Applied |
| Constant | 3.89*** | 3.86*** | 3.96*** |
|  | (0.10) | (0.09) | (0.09) |
|  |  |  |  |
| Sample Size | 3,869 | 3,869 | 3,869 |
| R-squared | 0.17 | 0.17 | 0.17 |

Robust standard errors in parentheses

*** p<0.01, ** p<0.05, * p<0.1


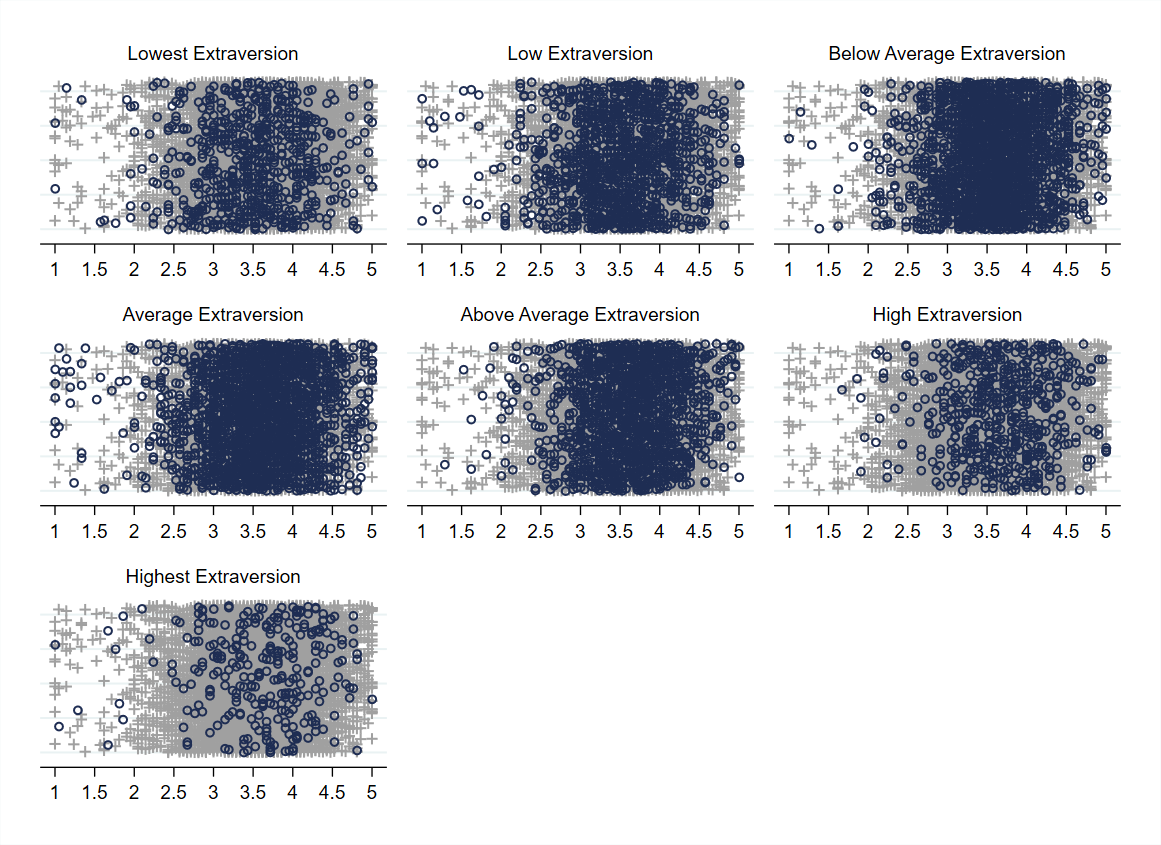


*Figure S1.* Distribution of Extraversion within the Compliance Scale


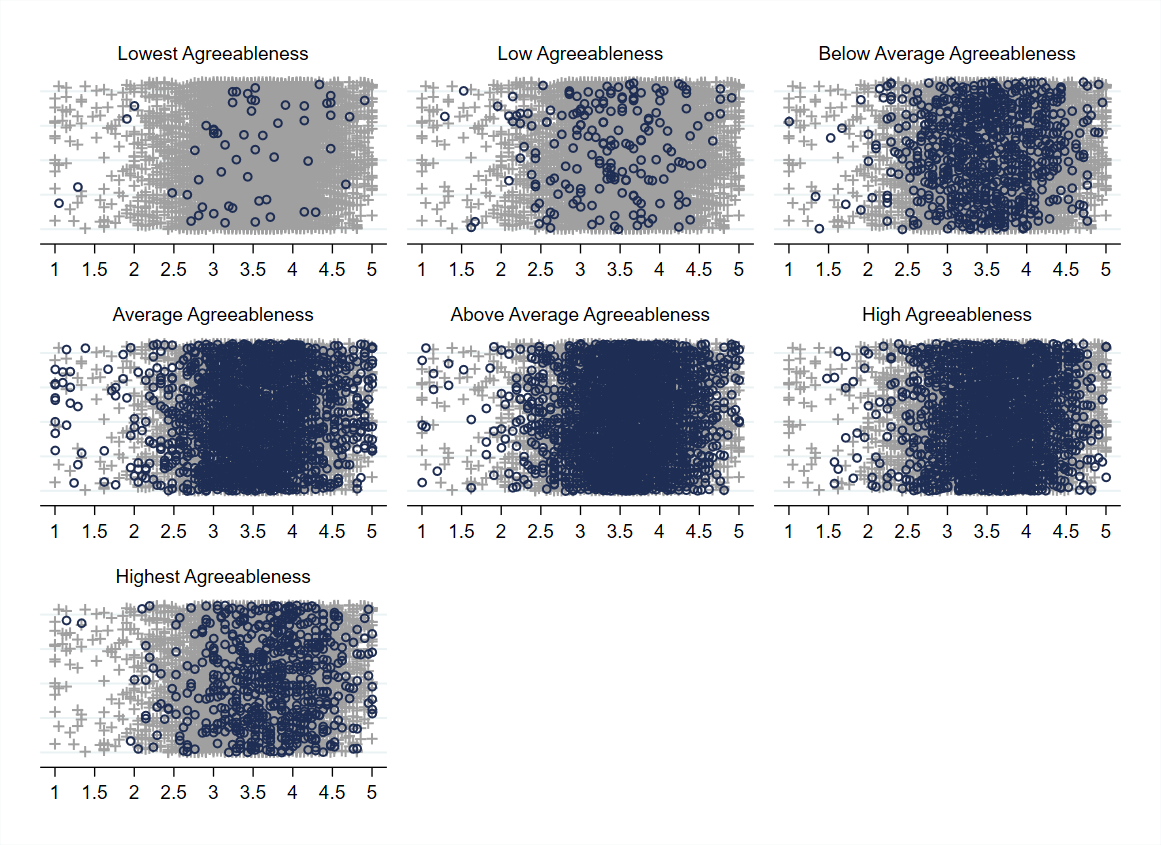


*Figure S2.* Distribution of Agreeableness within the Compliance Scale


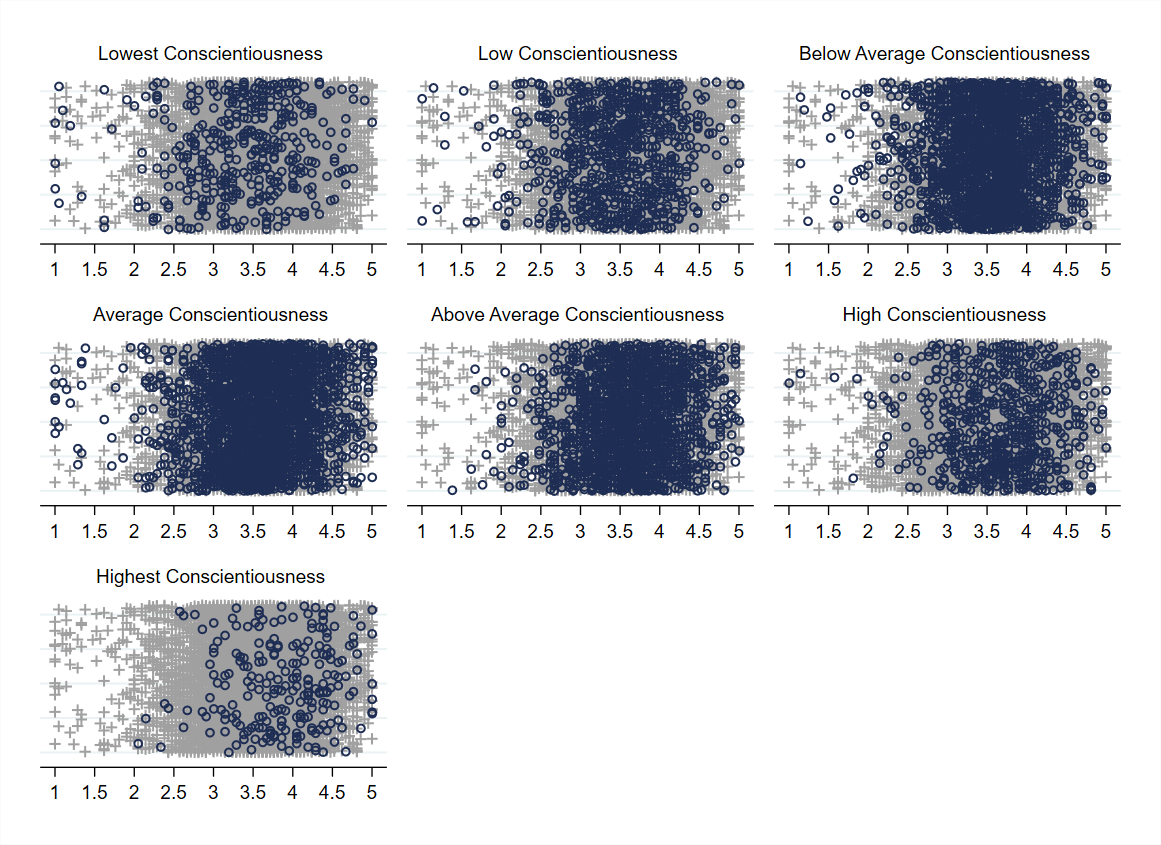


*Figure S3.* Distribution of Conscientiousness within the Compliance Scale


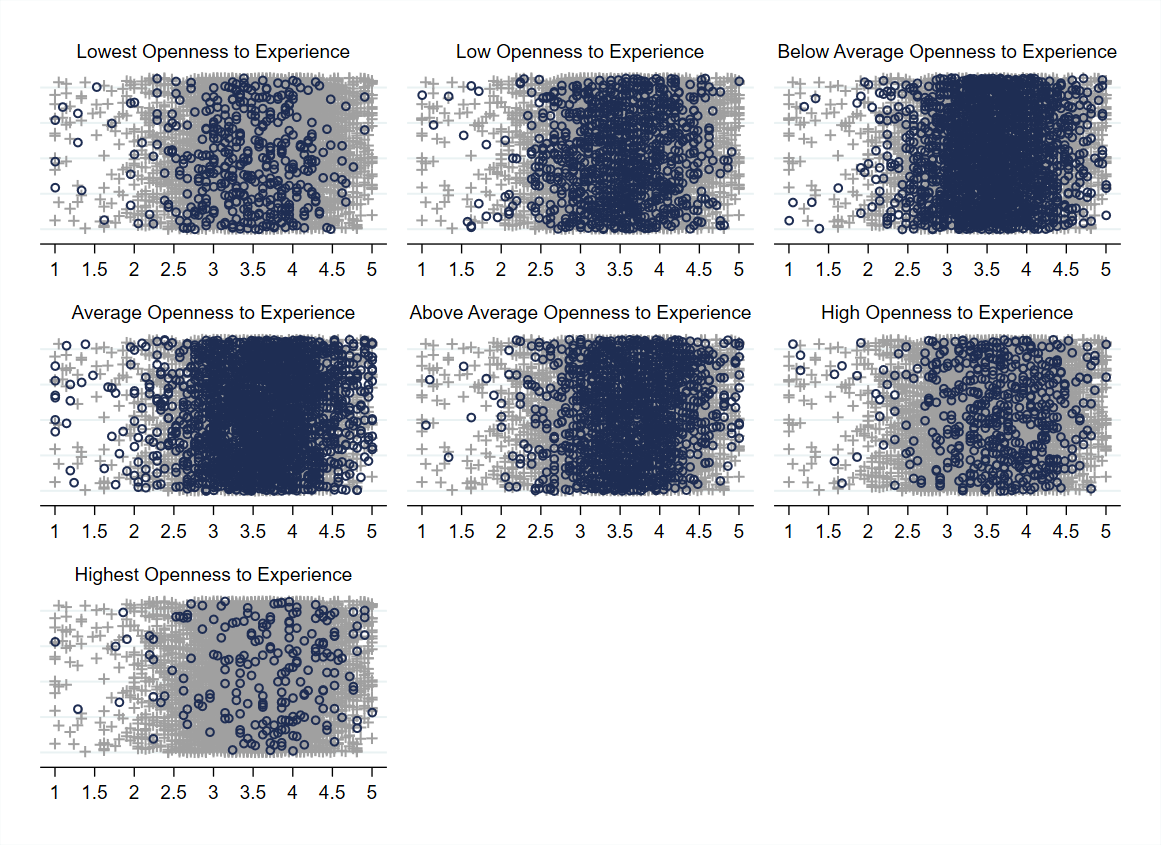


*Figure S4.* Distribution of Openness to Experience within the Compliance Scale


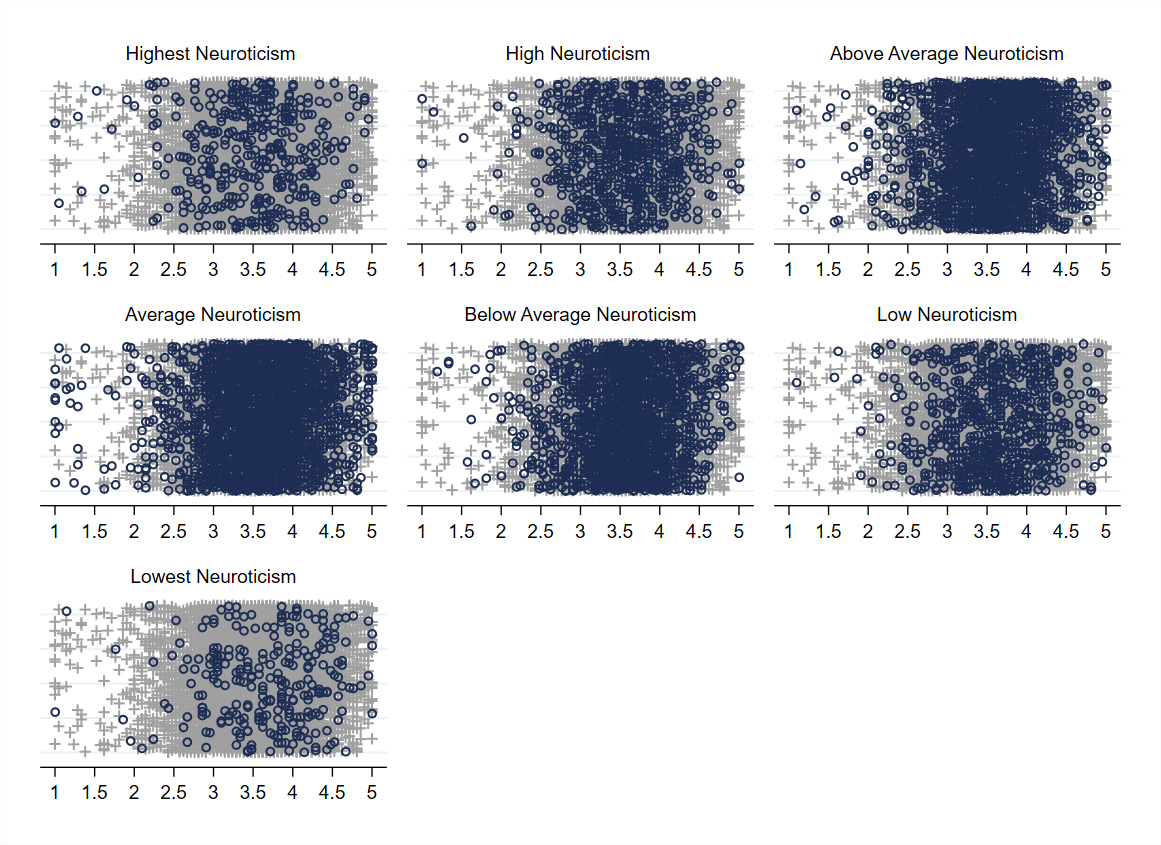


*Figure S5.* Distribution of Neuroticism within the Compliance Scale

**Stata Code**

*import delimited "118584-V1\rawdata.csv",*

*replace q3=. if q3==5*

*rename q3 conductmeasures*

*replace q4=. if q4==12*

*rename q4 actiondriver*

*tab work, gen(working)*

*replace q6s1=. if q6s1==6*

*replace q6s2=. if q6s2==6*

*replace q6s3=. if q6s3==6*

*gen actionstaken= (q5s1 + q5s2 + q5s3 + q5s4 + q5s5 + q5s6 + q5s7 + q5s8 + q5s9 + q5s10 + q5s11 + q5s12 + q5s13 + q5s14 + q5s15 + q5s16 + q5s17 + q5s18 + q5s19 + q5s20 + q5s21)/21*

*replace working3=. if working1==1*

*replace working3=. if working4==1*

*alpha q5s1 q5s2 q5s3 q5s4 q5s5 q5s6 q5s7 q5s8 q5s9 q5s10 q5s11 q5s12 q5s13 q5s14 q5s15 q5s16 q5s17 q5s18 q5s19 q5s20 q5s21*

*gen extraversion= 5-q7s1*

*gen extra= (extraversion+ q7s6)/2*

*gen agreeableness= 5-q7s7*

*gen agree= (agreeableness+ q7s2)/2*

*gen conscientiousness= 5-q7s3*

*gen consc= (conscientiousness+ q7s8)/2*

*gen openness= 5- q7s5*

*gen open= (openness+q7s10)/2*

*gen neuro= 5- q7s9*

*gen emostable= (neuro+q7s4)/2*

*gen actionstakenreverse= 6-actionstaken*

*asdoc pwcorr actionstakenreverse extra agree consc open emostable work sex age married univ hincome q1s1 q1s2 q1s3 q1s4 q1s5 q1s6 q1s7 q1s8 q1s9 q1s10 ,star(all) list replace nonum dec(2)*

*reg actionstakenreverse conductmeasuresreverse extra agree consc open emostable sex age married univ hincome q1s1 q1s2 q1s3 q1s4 q1s5 q1s6 q1s7 q1s8 q1s9 q1s10 , r*

*gen model=1 if e(sample)==1*

*tab model, missing*

*keep if model==1*

*su actionstakenreverse conductmeasuresreverse extra agree consc open emostable sex age married univ hincome q1s1 q1s2 q1s3 q1s4 q1s5 q1s6 q1s7 q1s8 q1s9 q1s10*

*generate id=_n*

*fabplot scatter id actionstakenreverse , by( extra )*

*fabplot scatter id actionstakenreverse , by( agree )*

*fabplot scatter id actionstakenreverse , by( consc )*

*fabplot scatter id actionstakenreverse , by( open )*

*fabplot scatter id actionstakenreverse , by( emostable )*

*regress actionstakenreverse extra agree consc open emostable sex married univ i.age i.hincome q1s1 q1s2 q1s3 q1s4 q1s5 q1s6 q1s7 q1s8 q1s9 q1s10 , r*

*meglm actionstakenreverse extra agree consc open emostable sex married univ i.age q1s1 q1s2 q1s3 q1s4 q1s5 q1s6 q1s7 q1s8 q1s9 q1s10 ||hincome:,*

*meglm actionstakenreverse extra agree consc open emostable sex married univ i.hincome q1s1 q1s2 q1s3 q1s4 q1s5 q1s6 q1s7 q1s8 q1s9 q1s10 ||age: ,*

*regress actionstakenreverse extra agree consc open emostable sex married univ i.age i.hincome q1s1 q1s2 q1s3 q1s4 q1s5 q1s6 q1s7 q1s8 q1s9 q1s10 , r*

*margins, at(extra=(1 2 3 4) ) asbalanced*

*marginsplot, name(model1)*

*margins, at(agree=(1 2 3 4) ) asbalanced*

*marginsplot, name(model2)*

*margins, at(consc=(1 2 3 4) ) asbalanced*

*marginsplot, name(model3)*

*margins, at(open=(1 2 3 4) ) asbalanced*

*marginsplot, name(model4)*

*margins, at(emostable=(1 2 3 4) ) asbalanced*

*marginsplot, name(model5)*

*graph combine model1 model2 model3 model4 model5,*

*For calculating the odds ratio using ordinal logit:*

*ologit actionstakenreverse extra agree consc open emostable sex married univ i.age i.hincome q1s1 q1s2 q1s3 q1s4 q1s5 q1s6 q1s7 q1s8 q1s9 q1s10 , or*
